# CaMKII Phosphorylation Regulates Synaptic Enrichment of Shank3

**DOI:** 10.1523/ENEURO.0481-20.2021

**Published:** 2021-05-22

**Authors:** Jaehoon Jeong, Yan Li, Katherine W. Roche

**Affiliations:** National Institute of Neurological Disorders and Stroke, National Institutes of Health, Bethesda, MD 20892

**Keywords:** autism, CaMKII, phosphorylation, posttranslational modification, Shank3

## Abstract

SHANK3 is a large scaffolding protein in the postsynaptic density (PSD) that organizes protein networks, which are critical for synaptic structure and function. The strong genetic association of *SHANK3* with autism spectrum disorder (ASD) emphasizes the importance of SHANK3 in neuronal development. SHANK3 has a critical role in organizing excitatory synapses and is tightly regulated by alternative splicing and posttranslational modifications. In this study, we examined basal and activity-dependent phosphorylation of Shank3 using mass spectrometry (MS) analysis from *in vitro* phosphorylation assays, *in situ* experiments, and studies with cultured neurons. We found that Shank3 is highly phosphorylated, and we identified serine 782 (S782) as a potent CaMKII phosphorylation site. Using a phosphorylation state-specific antibody, we demonstrate that CaMKII can phosphorylate Shank3 S782 *in vitro* and in heterologous cells on cotransfection with CaMKII. We also observed an effect of a nearby ASD-associated variant (Shank3 S685I), which increased S782 phosphorylation. Notably, eliminating phosphorylation of Shank3 with a S782A mutation increased Shank3 and PSD-95 synaptic puncta size without affecting Shank3 colocalization with PSD-95 in cultured hippocampal neurons. Taken together, our study revealed that CaMKII phosphorylates Shank3 S782 and that the phosphorylation affects Shank3 synaptic properties.

## Significance Statement

The precise regulation of scaffolding proteins is important for neuronal development and dysregulation underlies some neurodevelopmental disorders. As an excitatory synapse scaffolding protein, SHANK3 plays a critical role in synapse structure and function and has a strong genetic linkage to autism spectrum disorder (ASD). In this report, we examined the fine regulation of Shank3 by phosphorylation. Our study characterizes a CaMKII phosphorylation site on Shank3 (S782) *in vitro*, *in situ*, and in neurons. The Shank3 phosphorylation is modulated by a neighboring ASD-associated mutation. Furthermore, S782 phosphorylation is involved in Shank3 synaptic enrichment. These findings reveal molecular mechanisms of SHANK3 function at excitatory synapses and a potential role in ASD etiology.

## Introduction

Shank/ProSAP family proteins consist of three isoforms (SHANK1, SHANK2, and SHANK3) and help organize protein networks in the postsynaptic density (PSD; Ehlers, 1999; Sheng and Kim, 2000). Shank family proteins have five conserved domains: an ankyrin repeat domain (ANK), Src homology 3 (SH3) domain, a PSD-95/Discs large/ZO-1 (PDZ) domain, a proline-rich region, and a sterile α motif (SAM) domain (Sheng and Kim, 2000). More than 30 synaptic proteins, including receptors, ion channels, cytoskeletal proteins, signaling molecules, and enzymes, have been identified to interact with Shank3 (Kreienkamp, 2008). These interactions are mediated by the conserved domains in Shank3; and, because of the similarity of protein domains, many of the identified binding partners are shared among all Shank family proteins.

A variety of *SHANK3* gene defects have been revealed in patients with autism spectrum disorder (ASD; Jiang and Ehlers, 2013; Ruzzo et al., 2019; Satterstrom et al., 2020). Chromosomal disruptions of the *SHANK3* gene within the critical region of 22q13.3 deletion syndrome (Phelan–McDermid syndrome; PMS) have been widely studied (Wilson et al., 2003), and chromosomal microdeletions of *SHANK1* and *SHANK2* genes have also been reported (Pinto et al., 2010; Sato et al., 2012). Genome and exome sequencing studies have revealed inherited or *de novo* mutations associated with ASD. These point mutations include missense, nonsense, frame shift, and splice site mutations that disrupt SHANK3 protein expression. The ASD-associated point mutations are primarily found in the *SHANK3* gene, although cases identified in the *SHANK1* and *SHANK2* genes are also accumulating (Sato et al., 2012; Jiang and Ehlers, 2013).

Because SHANK3 plays such a critical role in synapse structure and function and because of its close genetic linkage to ASD, understanding the precise molecular mechanisms that regulate SHANK3 is important. Since SHANK3 is a huge molecular weight protein with multiple domains, people have mostly focused on characterizing the synaptic role of the interaction between the individual SHANK3 protein domains and their binding partners by using *in vitro* experiments combined with knock-in or knock-out animal models (Jiang and Ehlers, 2013). It is known that phosphorylation of scaffolding proteins is an important regulatory mechanism for protein interactions, subcellular distribution, and physiological functions (Iasevoli et al., 2013). For instance, PSD-95, an excitatory synapse scaffolding protein, has multiple phosphorylation sites, which are targeted by several kinases, such as JNK1, CDK5, and GSK3β, to modulate its synaptic accumulation and promote receptor clustering (Morabito et al., 2004; Kim et al., 2007; Nelson et al., 2013). More recently, the regulation of Shank3 by phosphorylation of one residue, serine 685 (S685) has been reported using a variety of techniques (Wang et al., 2020; Perfitt et al., 2020a). This particular site coincides with an ASD-associated variant site, and the phosphorylation and rare variant are associated with the recruitment of Abelson interactor 1 (ABI1) to Shank3 protein. These studies demonstrated S685 is phosphorylated in a transgenic mouse model (Wang et al., 2020), that CaMKII and PKA can phosphorylate Shank3 S685 *in vitro* (Wang et al., 2020; Perfitt et al., 2020a), and the phosphorylation is required for the ABI1 interaction with Shank3 (Wang et al., 2020; Perfitt et al., 2020a).

In this study, we characterized basal phosphorylation of Shank3 in heterologous cells and also on CaMKII activation. CaMKII is an abundant protein in neurons and involved in many critical regulatory pathways in the PSD (Hell, 2014). By conducting a series of mass spectrometry (MS) analyses with *in vitro*, *in situ*, and neuronal samples, we found Shank3 is highly phosphorylated under basal conditions and that the S782 residue on Shank3 is a potent CaMKII phosphorylation site. We demonstrated CaMKII directly phosphorylates Shank3 S782 by probing immunoblots with a S782 phosphorylation state-specific antibody. We did not observe any effects of Shank3 S782 phosphorylation on protein binding to the Shank3 PDZ domain. However, strikingly in hippocampal cultured neurons, we observed Shank3 S782 phosphorylation regulates Shank3 enrichment in synaptic spines and the size of PSD-95 defined synapses.

## Materials and Methods

### Plasmids and antibodies

HA-tagged rat Shank3 plasmid was kindly provided by Eunjoon Kim. CaMKII and CaMKII R42K plasmids were characterized in our previous studies. pClneo-GKAP-myc plasmids were used for biochemical and imaging experiments. The commercial antibodies used in this study were rabbit anti-Shank3 (Synaptic System, 162 302), mouse anti-PSD-95 (Neuromab, clone K28/43), mouse anti-myc (Cell Signaling, clone 9B11), rabbit anti-HA (Abcam, ab9110), rat anti-HA (Roche), rabbit anti-β tubulin (Sigma, T2200), and mouse anti-β actin (ABM, G043).

### MS

In-gel samples were digested with chymotrypsin at 25°C for 18 h. Peptides were extracted and desalted before being injected into a nano-liquid chromatography with tandem MS (LC/MS/MS) system where an Ultimate 3000 HPLC (Thermo-Dionex) was coupled to an Orbitrap Elite mass spectrometer (Thermo Scientific) via an Easy-Spray ion source (Thermo Scientific). Peptides were separated on a ES802 Easy-Spray column (75-μm inner diameter, 25 cm in length, 3-μm C18 beads; Thermo Scientific) with a 25-min linear gradient of 2–27% mobile phase B (mobile phase A: 2% acetonitrile, 0.1% formic acid; mobile phase B: 98% acetonitrile, 0.1% formic acid). The HPLC flow rate was 300 nl/min.

Thermo Scientific Orbitrap Elite mass spectrometer was operated in positive data-dependent LC-MS/MS mode. The resolution of the survey scan was set at 60k at m/z 400. The m/z range for MS scans was 300–1600. For MS/MS data acquisition, the decision-tree mode was activated, the minimum signal intensity required to trigger MS/MS scan was 1e4, the top ten most abundant ions were selected for product ion analysis, the isolation width was 1.9 m/z, and the dynamic exclusion window was 9 s.

Xcalibur RAW files were converted to peak list files in mgf format using Mascot Distiller. Database search was performed using Mascot Daemon (2.4.0) against NCBInr_Human database. Spectra of phosphopeptides were manually checked.

### GST fusion protein production

Shank3 fragments were subcloned into the pGEX-4T plasmid and transformed to BL21 bacterial cells. Bacterial cultures were grown at 37°C to an absorbance at 600 nm of 1.1–1.2 of the culture media; 50 μm isopropyl β-d-1-thiogalactopyranoside (IPTG) was added to the cultures and incubated at 16°C overnight to induce fusion protein expression. The bacterial pellets were then lysed in a Tris-buffered saline (TBS) buffer containing protease inhibitors (Roche), 100 μg/ml lysozyme, 15 mm dithiothreitol (DTT), 10 mm EDTA, and 1.5% Sarkosyl. The sonicated lysate was neutralized with Triton X-100 to a final concentration of 4%. The lysates were incubated with glutathione–Sepharose 4B (GE Healthcare) for 1 h at 4°C and subsequently washed with TBS buffer containing 1 mm EDTA and 0.1% Triton X-100.

### *In vitro* kinase assay and GST-pulldown

GST fusion proteins were phosphorylated in 10 mm HEPES (pH 7.0), 20 mm MgCl_2_, 50 μm ATP, and 1 pmol of [γ-^32^P] ATP (3000 Ci mmol^−1^) with 50 ng of purified CaMKII catalytic subunit (Promega). *In vitro* kinase assays were performed at 30°C for 15 min. Proteins were eluted from the glutathione-Sepharose resin and resolved by SDS-PAGE and analyzed by immunoblotting.

For GST-pulldown, the cell lysates were prepared in hypoosmotic buffer (20 mm Tris-HCl, pH 8.8, 5 mm EDTA, and 1% DOC). GST fusion proteins were incubated with the lysate at 4°C overnight. The samples were resolved by SDS-PAGE and analyzed by immunoblotting.

### Transfection and immunoblot

HEK293 cells were transfected with Lipofectamine^2000^ and 2 d after transfection the cells were lysed in a TBS buffer containing 150 mm NaCl, 50 mm Tris-HCl (pH 8.0), 1 mm EDTA, 1% Triton X-100. Lysates were centrifuged at 16,000 × *g* for 15 min at 4°C and supernatant were used for the analysis. For coimmunoprecipitation, lysates were incubated with an appropriate antibody at 4°C overnight and protein-A-Sepharose beads (GE Healthcare) at 4°C for 1 h. All chemiluminescence blots were captured with a ChemiDoc Imaging System (Bio-Rad).

### Neuronal cultures

For biochemical analyses and immunocytochemistry experiments, we used primary cultured cortical and hippocampal neurons from embryonic day (E)18 Sprague Dawley rats of either sex. Briefly, embryonic hippocampal or cortical tissues were dissociated at 37°C for 30 min by 0.05% trypsin in 10 mm HBSS containing 1.37 mg/ml DNase I. Neurons were plated and maintained in serum-free Neurobasal Medium supplemented with 2% (v/v) B-27 and 2 mm L-glutamine. We adhered to the guidelines of the National Institutes of Health Animal Care and Use Committee regarding the care and use of animals for this study (protocol #1171).

### Immunocytochemistry

Cultured hippocampal neurons were grown on glass coverslips precoated with poly-D-lysine (Sigma). Neurons were transfected with the indicated plasmids with Lipofectamine^2000^ at 12–15 days *in vitro* (DIV) and prepared for analysis at DIV19–DIV21, if not described otherwise.

To label HA-Shank3, the transfected neurons were fixed with 4% paraformaldehyde and 4% sucrose in PBS for 10 min. After fixation, the neurons were permeabilized with 0.25% Triton X-100 in PBS and blocked with 10% goat serum. The neurons were labeled with rat anti-HA antibody and Alexa Fluor 555-conjugated anti-rat secondary antibody (Invitrogen). For endogenous PSD-95 and Shank3 staining, neurons were prepared as above, then labeled with anti−PSD-95 (NeuroMab, clone K28/43) and anti-Shank3 (Synaptic System, 162302).

For analysis, regions from three dendrites per each neuron were collected and quantified by the fluorescence intensity of target proteins. All images were captured with a 63× objective on a Zeiss LSM 510 confocal microscope and analyzed with the ImageJ and MetaMorph version 7.

### Experimental design and statistical analyses

Data analysis was conducted in Image Lab Software (Bio-Rad) or GraphPad Prism (GraphPad Software).

## Results

### Shank3 is a highly phosphorylated protein

**Figure 1.**
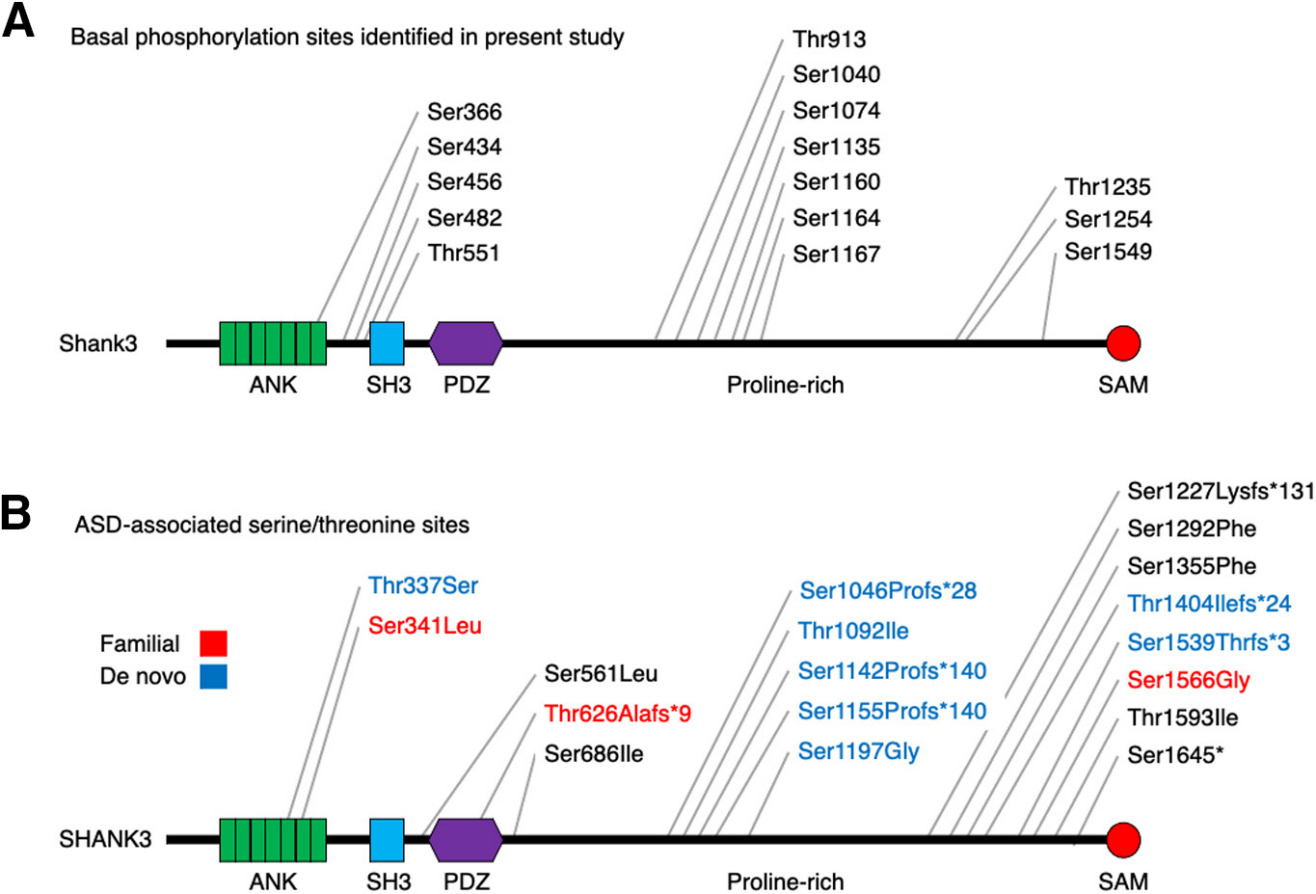
Shank3 is a highly phosphorylated protein. ***A***, Basal phosphorylation sites in full-length Shank3 protein identified by MS illustrated on a schematic drawing. HA-Shank3 was expressed in HEK293 cells and immunoprecipitated HA-Shank3 was subjected to MS analysis. ***B***, ASD-associated serine/threonine variants found in annotated public databases are depicted on a schematic drawing. Familial variants are indicated in red, and *de novo* variants are indicated in blue. Variants with unknown inheritance pattern are in black.

Phosphorylation is one of the most common regulatory mechanisms to modulate synaptic protein interactions and subcellular localization. Since SHANK3 is a large essential scaffolding protein in the PSD, we sought to investigate phosphorylation-mediated SHANK3 regulation. To establish the SHANK3 phosphorylation profile, we expressed HA-Shank3 in HEK293 cells, and HA-Shank3 was immunoprecipitated for MS analysis. In the MS results, 87% of the Shank3 protein sequence was recovered and, notably, multiple basal phosphorylation sites were observed (Fig. 1*A*). Multiple phosphorylation sites on the Shank3 protein have been previously reported in several studies by analyzing various kinds of samples (Thomas et al., 2005; Huttlin et al., 2010; Trinidad et al., 2012; Nagai et al., 2016; Wang et al., 2020). However, because of its large gene and protein size, consequences of these phosphorylation events for Shank3 function have been rarely identified at the molecular and cellular levels (Wang et al., 2020).

We next compared the Shank3 basal phosphorylation sites with ASD-associated serine/threonine variants in annotated public databases to find any ASD-associated phosphorylation site. Since *SHANK3* is the most well studied ASD-associated gene, 249 rare variants are found in databases (https://gene.sfari.org/database/human-gene/SHANK3). Among these variants, 11 missense and 7 frameshift variants occurred on serine/threonine sites, which might lead to disruption of serine/threonine phosphorylation (Fig. 1*B*). Other than S686 (S685 in rodent; Wang et al., 2020), we did not find any overlap between the ASD-associated *SHANK3* serine/threonine variants and identified Shank3 phosphorylation sites in phosphoproteomic databases (https://www.phosphosite.org/proteinAction.action?id=14451&showAllSites=true) and our Shank3 basal phosphorylation sites (Fig. 1*A*,*B*). Thus, to our surprise, we did not identify ASD-associated SHANK3 serine/threonine phosphorylation that are also in ASD databases. It could be that this negative correlation results from differing protocols in collecting samples or limited Shank3 protein coverage for phospho-proteomic analysis, but thus far the datasets are distinct.

### CaMKII phosphorylates Shank3 on S782

**Figure 2.**
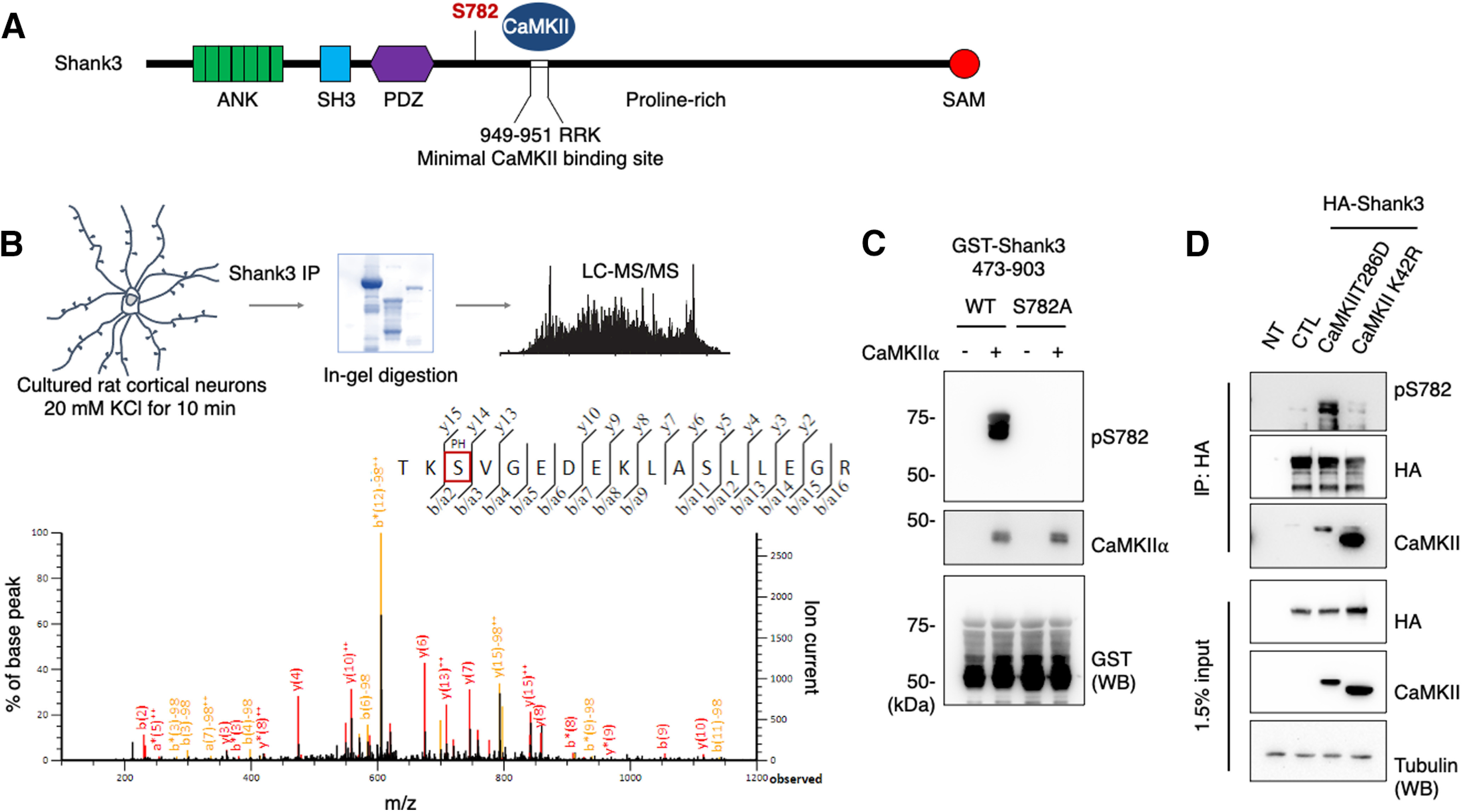
CaMKII phosphorylates Shank3 on S782. ***A***, Schematic figures denote CaMKII-dependent phosphorylation on Shank3 S782. ***B***, Cultured rat cortical neurons were treated with 20 mM KCl for 10 min, and endogenous Shank3 was enriched by immunoprecipitation using anti-Shank3 antibody. Protein samples were resolved by SDS-PAGE. Samples were in-gel digested and analyzed using the LC/MS/MS method. Representative MS/MS spectrum of the phosphorylated Shank3 peptide found in the samples from cultured rat cortical neurons treated with KCl. MS/MS spectrum from *in vitro* phosphorylation assays and *in situ* experiments are presented in Extended Data Figure 2-1. ***C***, Immunoblot analysis of GST-Shank3 (473–903) WT and S782A fusion proteins that were phosphorylated *in vitro* with purified CaMKII and probed with pS782 antibody. ***D***, HA-Shank3 was transfected or cotransfected with constitutively active CaMKII T286D or constitutively inactive CaMKII K42R in HEK293 cells. The Shank3 pS782 antibody immunoreactivity in the immunoprecipitates with HA antibody were analyzed by immunoblotting.

As we observed multiple basal phosphorylation sites on Shank3, we next investigated whether CaMKII phosphorylates Shank3. CaMKII is the most abundant synaptic kinase, and we therefore decided to characterize it first. To identify CaMKII phosphorylation sites on Shank3, we performed a series of MS analyses. First, we incubated GST-Shank3 fusion proteins, which cover regions of the Shank3 protein from amino acids 1–1265, with purified CaMKII and ATP for *in vitro* kinase assays. Next, full-length HA-Shank3 and CaMKII were expressed in HEK293 cells, and HA-Shank3 was immunoprecipitated using anti HA antibody. Lastly, we treated cultured rat cortical neurons with KCl to increase endogenous CaMKII activity (Bemben et al., 2014), and endogenous Shank3 were enriched from the neuron lysates by immunoprecipitation using anti-Shank3 antibody. In each of these three experimental paradigms, the isolated protein was analyzed using MS. Figure 2*B* shows a schematic of the experimental procedure for the analysis of the samples from the cultured neurons and its representative MS/MS spectrum (Fig. 2*B*). In these MS analyses, we found phospho-peptides corresponding to phosphorylation on Shank3 S782 from all different types of samples (Fig. 2; Extended Data Fig. 2-1*C*,*D*). These results show a reliability of Shank3 S782 phosphorylation by CaMKII (Fig. 1*A*). We also observed the Shank3 S685 phosphorylation (De Rubeis et al., 2014; Wang et al., 2020) from the *in vitro* kinase assay samples (Extended Data Fig. 2-1*B*) but not from the cell line and cultured neuron samples.

10.1523/ENEURO.0481-20.2021.f2-1 Extended Data Figure 2-1 MS analysis from *in vitro* phosphorylation assays and *in situ* experiments. ***A***, A schematic of the experimental procedure for the analysis of the samples from *in vitro* phosphorylation assays and *in situ* experiments. ***B***, ***C***, Representative MS/MS spectra of the Shank3 S685 and S782 phosphorylated peptide found in the samples from *in vitro* phosphorylation assays. ***D***, Representative MS/MS spectrum of the Shank3 S782 phosphorylated peptide found in the samples from *in situ* experiments. Download Figure 2-1, TIF file.

To examine the phosphorylation of Shank3 S782 further, we generated a phosphorylation state-specific antibody, pS782 antibody. GST-Shank3 (473–903) wild-type (WT) or S782A fusion proteins were incubated with CaMKII and ATP *in vitro*. The samples were resolved by SDS-PAGE and analyzed by immunoblotting with the pS782 antibody. We observed CaMKII activity-dependent immunoreactivity for GST-Shank3 WT and the serine to alanine mutation completely eliminated the immunoreactivity (Fig. 2*C*). To determine whether full-length Shank3 can be phosphorylated by CaMKII, we cotransfected HA-Shank3 and constitutively active CaMKII (CaMKII T286D) or inactive CaMKII (CaMKII R42K) in HEK293 cells. HA-Shank3 was immunoprecipitated using HA antibody, and the immunoprecipitates were resolved by SDS-PAGE and analyzed by immunoblotting. The immunoblots showed that both active and inactive CaMKII are coimmunoprecipitated with HA-Shank3 as shown previously (Perfitt et al., 2020b), but the pS782 immunoreactivity for HA-Shank3 was specifically increased on active CaMKII cotransfection, which was not the case for the inactive CaMKII R42K cotransfection (Fig. 2*D*). Notably, the CaMKII binding motif, R^949^-R^950^-K^951^ (Perfitt et al., 2020b), has been characterized, and the Shank3 S782 phosphorylation site is not far from the binding motif, further implicating CaMKII regulation (Fig. 2*A*). All of these results indicate that CaMKII regulates Shank3 via direct phosphorylation on S782.

### An ASD-associated Ser685Ile mutation increases CaMKII-mediated Shank3 S782 phosphorylation

**Figure 3.**
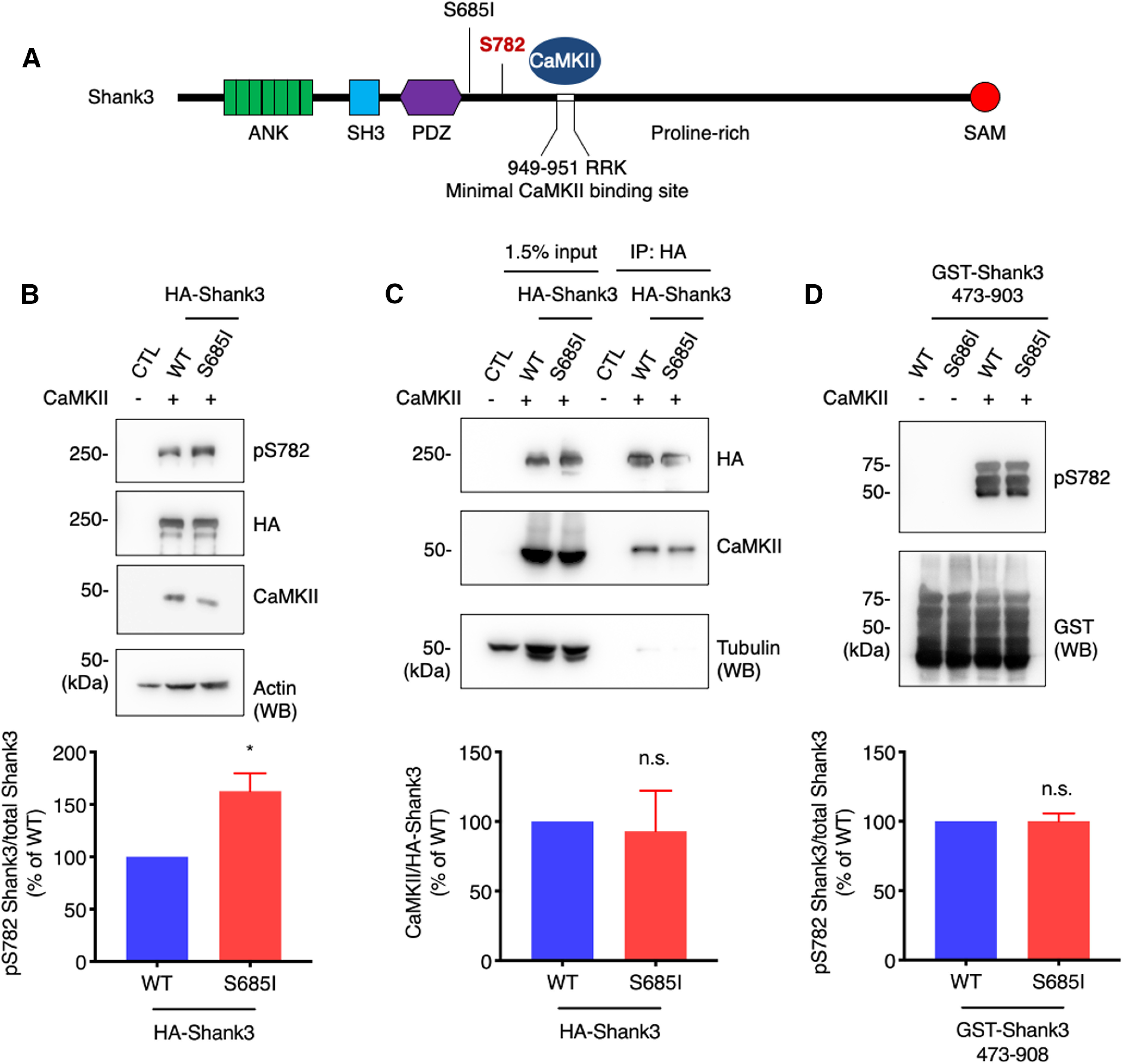
An ASD-associated Shank3 S685I mutation increases CaMKII-mediated Shank3 S782 phosphorylation. ***A***, Schematic figures denote the ASD-associated Ser685Ile (S685I) mutation and CaMKII-dependent phosphorylation on Shank3 S782. ***B***, HA-Shank3 WT or S685I were cotransfected with constitutively active CaMKII T286D in HEK293 cells. The Shank3 pS782 antibody immunoreactivity was analyzed by immunoblotting. Quantitative graph represents mean ± SEM (*n* = 4); *p = 0.0102 using an unpaired *t* test. ***C***, HA-Shank3 WT or S685I were cotransfected with constitutively active CaMKII T286D in HEK293 cells. HA-Shank3 was immunoprecipitated with HA antibody and CaMKII T286D was analyzed by immunoblotting. Quantitative graph represents mean ± SEM (*n* = 4); statistical significance was calculated using an unpaired *t* test. n.s., not significant. ***D***, Immunoblot analysis of GST-Shank3 473–903 WT or S685I fusion proteins that were phosphorylated *in vitro* with purified CaMKII and probed with pS782 and GST antibody. Quantitative graph represents mean ± SEM (*n* = 3), and statistical significance was calculated using an unpaired *t* test. n.s., not significant.

Noticeably, Shank3 S782 is in close proximity to an ASD-associated Ser686Ile (S686I) missense variant (De Rubeis et al., 2014; Fig. 3*A*). Therefore, we next asked whether the S686I (S685I in rodent) mutation affects Shank3 S782 phosphorylation. To answer this question, we cotransfected HA-Shank3 (WT or S685I) and constitutively active CaMKII (CaMKII T286D) in HEK293 cells. The cell lysates were resolved by SDS-PAGE and analyzed by immunoblotting. Interestingly, the immunoblots showed that the pS782 immunoreactivity for HA-Shank3 S685I was increased compared with HA-Shank3 WT (Fig. 3*B*). We were curious whether the increased S782 phosphorylation on HA-Shank3 S685I is because of an enhanced CaMKII interaction. To examine this, we cotransfected HA-Shank3 WT or S685I and constitutively active CaMKII in HEK293 cells. HA-Shank3 were immunoprecipitated using HA antibody, and coimmunoprecipitated CaMKII was analyzed by immunoblotting. The immunoblots showed that CaMKII binding with HA-Shank3 S685I was not altered compared with HA-Shank3 WT (Fig. 3*C*). To explore further, GST-Shank3 (473–903) WT or S685I fusion proteins that lack the CaMKII binding site were incubated with CaMKII and ATP *in vitro*. The samples were resolved by SDS-PAGE and analyzed by immunoblotting with pS782 antibody. We observed that the pS782 immunoreactivity for GST-Shank3 473–903 WT and S685I are comparable (Fig. 3*D*). These results indicate that *in situ* context is important for the Shank3 S782 phosphorylation.

### Shank3 S782 phosphorylation does not affect PDZ domain binding

Many studies have shown that the SHANK3 PDZ domain is important for protein interactions with several binding partners, such as GKAPs, PSD-93, mGluR1/5, and PLC-β3 (Sheng and Kim, 2000; Jiang and Ehlers, 2013). Since the PDZ interactions can be regulated by phosphorylation and S782 is ∼100 amino acids away from the PDZ domain, we tested whether S782 phosphorylation affects the Shank3 interaction with its binding partners.

**Figure 4.**
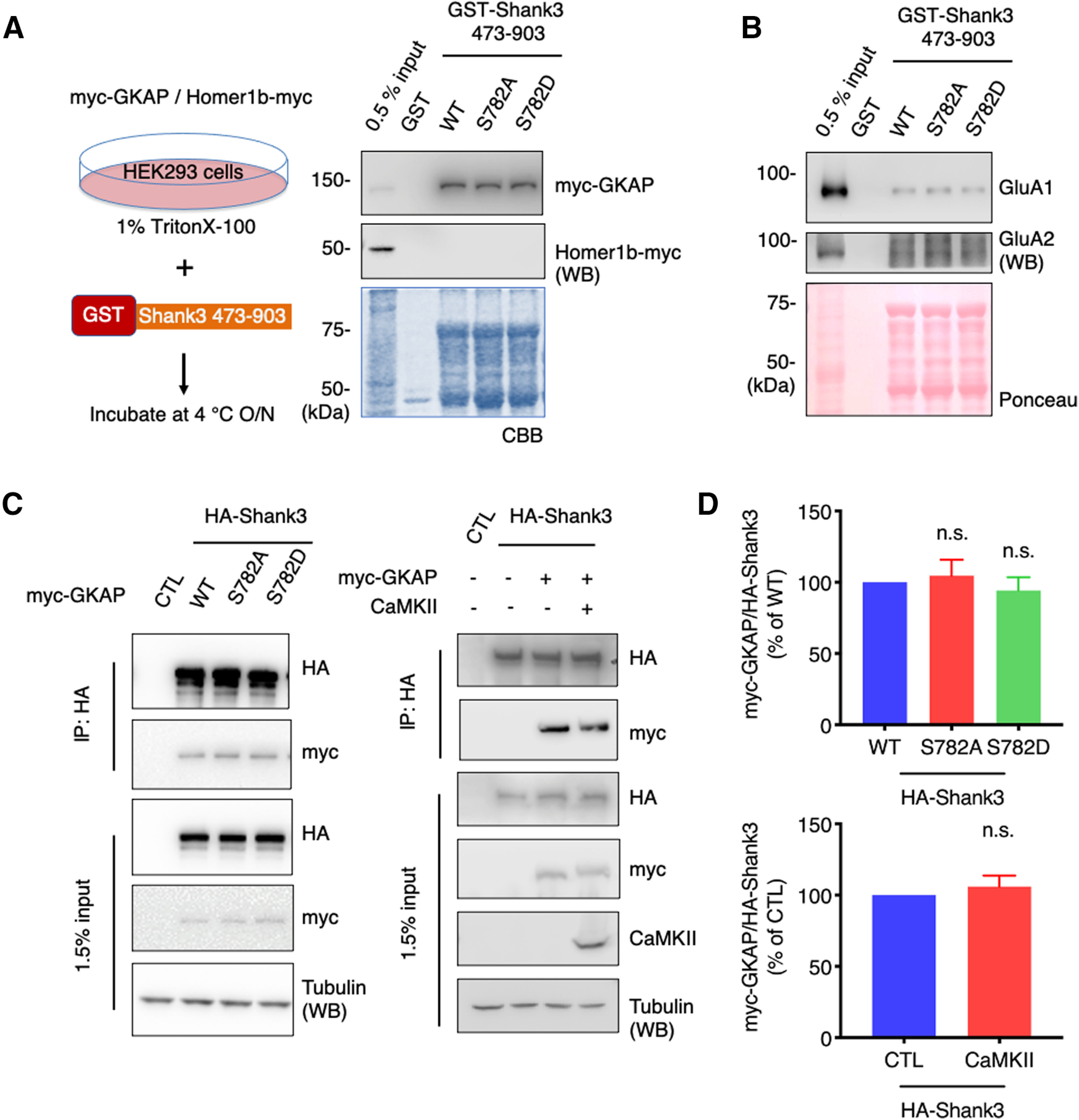
Shank3 S782 phosphorylation does not affect PDZ domain binding. ***A***, myc-GKAP or Homer1b-myc were expressed in HEK293 cells and the cell lysates were incubated with GST-Shank3 fusion proteins (WT, S782A, or S782D) for pulldown assays. myc-GKAP or Homer1b-myc in the pulldown sample were analyzed by immunoblotting. ***B***, GST-Shank3 fusion proteins (WT, S782A, or S782D) were incubated with rat brain P2 lysates for pulldown assays. The pulldown samples were analyzed by immunoblotting with indicated antibodies. ***C***, HA-Shank3 (WT, S782A, or S782D) and myc-GKAP were cotransfected in HEK293 cells (left). HA-Shank3 and myc-GKAP were cotransfected with or without CaMKII in HEK293 cells (right). HA-Shank3 was immunoprecipitated with HA antibody and myc-GKAP was analyzed by immunoblotting. ***D***, Quantitative graphs represent mean ± SEM (*n* = 3), and statistical significance was calculated using one-way ANOVA with Dunnett’s multiple comparison test (top) or an unpaired *t* test (bottom). n.s., not significant.

We expressed myc-GKAP in HEK293 cells and the cell lysates were incubated with GST-Shank3 (473–903) fusion proteins (WT, S782A, or S782D) for pulldown assays. Because GST-Shank3 (473–903) fusion proteins do not contain a Homer binding region, we used Homer1b-myc as a negative control. The isolated beads were resolved by SDS-PAGE and analyzed by immunoblotting. The amount of GST fusion proteins was visualized by Coomassie Brilliant Blue staining. Contrary to our expectations, neither S782A nor S782D showed changes compared with WT in binding with myc-GKAP (Fig. 4*A*). AMPAR subunits are also known to interact with Shank3 via its PDZ domain (Uchino et al., 2006; Jiang and Ehlers, 2013). To examine this, we incubated GST-Shank3 (473–903) fusion proteins (WT, S782A, or S782D) with rat brain P2 lysates for pulldown assays. The isolated beads were resolved by SDS-PAGE and analyzed by immunoblotting. The amount of GST fusion proteins was visualized by Ponceau staining on the same membrane. We did not observe any changes in binding with endogenous GluA1/2 receptors (Fig. 4*B*).

We reexamined the pulldown results using coimmunoprecipitation in intact cells. We cotransfected HA-Shank3 (WT, S782A, or S782D) and myc-GKAP in HEK293 cells. HA-Shank3 were immunoprecipitated from the cell lysates and resolved by SDS-PAGE, and coimmunoprecipitated myc-GKAP was analyzed by immunoblotting. We did not observe any significant differences in HA-Shank3 (WT, S782A, or S782D) and myc-GKAP interactions (Fig. 4*C*, left, *D*, top). To evaluate potential effects of cellular phosphorylation by CaMKII, we cotransfected HA-Shank3 WT and myc-GKAP with or without constitutively active CaMKII in HEK293 cells. We did not see any changes in HA-Shank3 and myc-GKAP interactions on CaMKII transfection (Fig. 4*C*, right, *D*, bottom). All these results indicate that Shank3 S782 phosphorylation is not necessarily associated with the Shank3 PDZ interactions.

### Shank3 S782 phosphorylation affects Shank3 enrichment and synapse size

**Figure 5.**
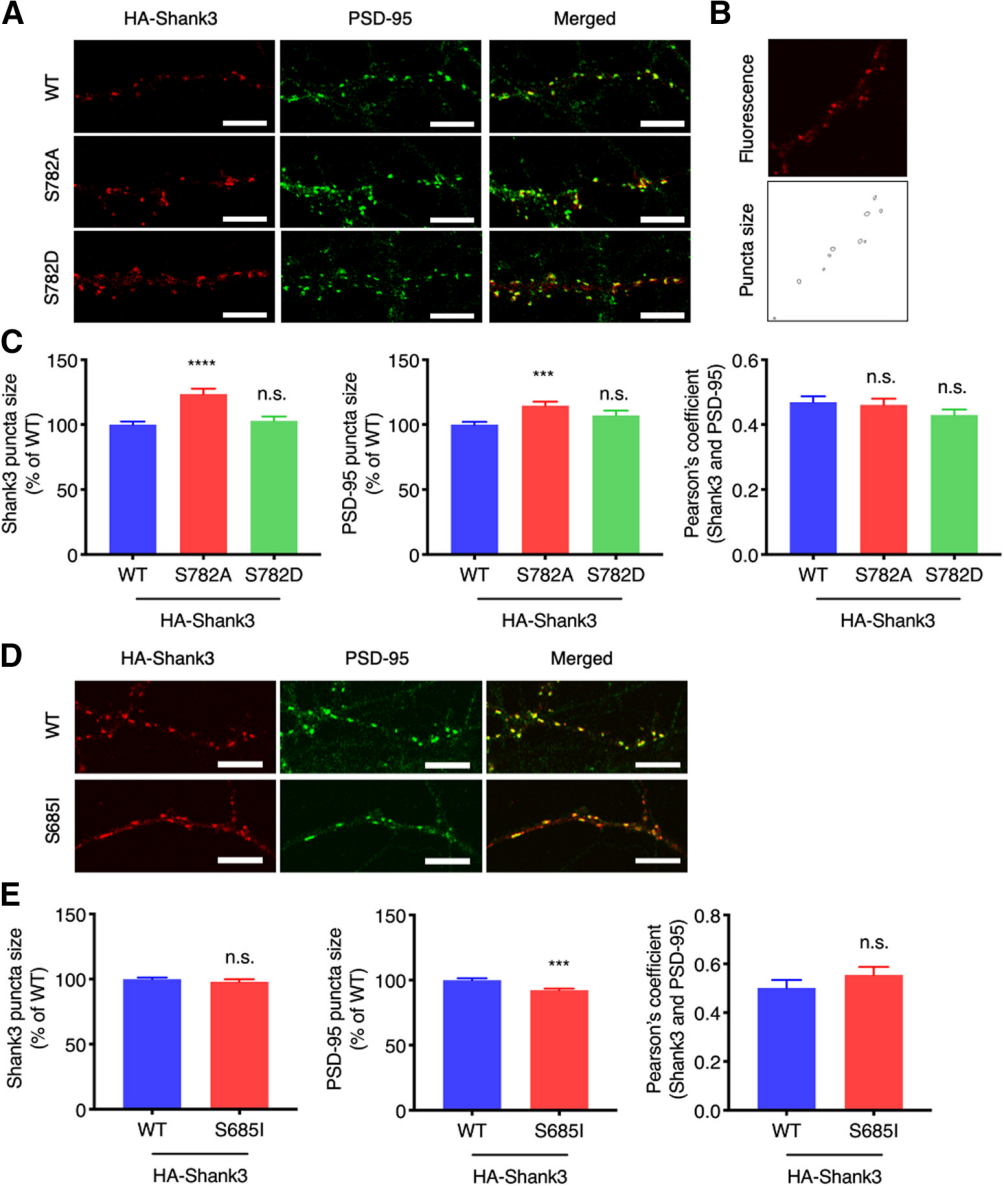
Shank3 S782 phosphorylation affects Shank3 enrichment and synapse size. ***A***, HA-Shank3 (WT, S782A, or S782D) was expressed in cultured rat hippocampal neurons. HA-Shank3 was stained with anti-HA and Alexa Fluor 555-conjugated secondary antibody (red). Endogenous PSD-95 was labeled with anti-PSD-95 antibody and Alexa Fluor 488-conjugated secondary antibody (green). Region from the secondary dendrites is shown. Scale bar: 5 μm. ***B***, Fluorescence signal was adjusted to measure perimeter of synaptic puncta. ***C***, Region from the secondary dendrites were analyzed for puncta size and Pearson’s coefficient. Graph indicates mean ± SEM (*n* = 28 for WT, *n* = 26 for S782A and S782D); ****p < 0.0001 and ***p < 0.0002 using one-way ANOVA with Dunnett’s multiple comparison test. Colocalization analysis between endogenous Shank3 and PSD-95 on CaMKII pharmacological activation in neurons are presented in Extended Data Figure 5-1. ***D***, HA-Shank3 (WT or S685I) was expressed in cultured rat hippocampal neurons. HA-Shank3 and endogenous PSD-95 were stained and analyzed as above. Region from the secondary dendrites is shown. Scale bar: 5 μm. ***E***, Graph indicates mean ± SEM (*n* = 24 for WT, *n* = 30 for S685I); ***p < 0.0002 using an unpaired *t* test. n.s., not significant.

To examine any effect of Shank3 S782 phosphorylation on synaptic properties, cultured rat hippocampal neurons were transfected with HA-Shank3 (WT, S782A, or S782D) at DIV12 and the neurons were prepared to label HA-Shank3 and endogenous PSD-95 at DIV20 for immunofluorescence confocal microscopy. Images of the secondary dendritic regions were captured, and the fluorescence signal was adjusted to measure the perimeter of synaptic puncta (Fig. 5*A*,*B*). We compared HA-Shank3 S782A and S782D to HA-Shank3 WT to investigate the site-specific effect on synapses. Interestingly, the phosphodeficient mutant, HA-Shank3 S782A, showed increased synaptic puncta size and endogenous PSD-95 puncta size in the HA-Shank3 S782A expressing dendrites was also increased (Fig. 5*C*). Colocalization of HA-Shank3 S782A or S782D with endogenous PSD-95 was not diminished compared with HA-Shank3 WT (Fig. 5*C*).

10.1523/ENEURO.0481-20.2021.f5-1 Extended Data Figure 5-1 No detectable effect of CaMKII pharmacological activation on the colocalization between endogenous Shank3 and PSD-95 in neurons. ***A***, ***B***, Cultured rat cortical neurons were treated with 20 μm KCl for 10 min to activate endogenous CaMKII. Endogenous Shank3 was stained with anti-Shank3 and Alexa Fluor 555-conjugated secondary antibody (red). Endogenous PSD-95 was labeled with anti-PSD-95 antibody and Alexa Fluor 488-conjugated secondary antibody (green). Regions from the secondary dendrites are shown. Scale bar: 10 μm. ***C***, Regions from the secondary dendrites were analyzed for Pearson’s coefficient. Graph indicates mean ± SEM (*n* = 10 for CTL, *n* = 10 for KCl treatment). Statistics using an unpaired *t* test. n.s., not significant. Download Figure 5-1, TIF file.

We showed that the ASD-associated Shank3 S685I mutation increases Shank3 S782 phosphorylation (Fig. 3*B*). To better understand the functional correlation between these two sites, we further examined Shank3 S685I synaptic enrichment and colocalization with PSD-95. Cultured rat hippocampal neurons were transfected with HA-Shank3 (WT or S685I) at DIV12 and prepared and analyzed as above. We observed that HA-Shank3 S685I shows comparable synaptic puncta size and colocalization with PSD-95 compared with HA-Shank3 WT (Fig. 5*D*,*E*). In contrast, PSD-95 puncta size was decreased in the HA-Shank3 S685I expressing dendrites (Fig. 5*D*,*E*), which might partially be the result of impaired synaptic protein recruitment by the Shank3 S685I mutant (Wang et al., 2020).

All these results indicate that Shank3 S782 phosphorylation regulates Shank3 synaptic puncta size as well as associated PSD-95 puncta size, without affecting colocalization of Shank3 and PSD-95.

## Discussion

*SHANK3* has been the focus of intense investigation because of its role in neurodevelopmental disorders such as ASD and PMS. The characterization of *SHANK3* has revealed a high degree of complexity as it exists in many forms because of alternative splicing and different start sites resulting in a variety of mature proteins. A recent study (Wang et al., 2020) has shown that Shank3 also have a high level of regulation by phosphorylation *in vitro*, although the relevant kinases and effect of synaptic activity on these phosphorylation events were not explored. In the current study, we used multiple experimental preparations to investigate the regulation of Shank3 by phosphorylation. Using MS analyses, we found that full-length Shank3 expressed in HEK293 cells is highly phosphorylated under basal conditions (Fig. 1). Our studies are in agreement with several previous studies that reported phosphorylation sites on Shank3. We initially hypothesized that identified phosphorylation would have considerable overlap with identified disease-associated variants based on one such example in the literature (S685; De Rubeis et al., 2014; Wang et al., 2020). However, surprisingly, we found the ASD variants did not overlap with any of the reported 55 phosphorylation sites in human *SHANK3*, except the single case.

We explored the relevant kinases targeting SHANK3. Because CaMKII is an abundant kinase in neurons and is involved in many critical synaptic events (Hell, 2014), we examined whether there was additional phosphorylation of Shank3 on CaMKII activation. We demonstrated CaMKII phosphorylation of Shank3 S782 using MS analysis of three different experimental samples: *in vitro* CaMKII assays with GST-Shank3 fusion proteins, immunoprecipitated HA-Shank3 from CaMKII expressing HEK293 cells, and enriched endogenous Shank3 from KCl-stimulated cultured cortical neurons (Fig. 2). We cannot completely exclude the possibility of S782 phosphorylation by other synaptic kinases in neurons, but we compared basal HA-Shank3 phosphorylation to CaMKII activated HA-Shank3 phosphorylation in the mass results from cell lines. The only other study reporting a direct phosphorylation of Shank3 by CaMKII involved *in vitro* assays assessing phosphorylation of GST-Shank3 (572–691) fusion proteins showing that Shank3 S685 can be phosphorylated by CaMKII (Perfitt et al., 2020a). Because this study looked specifically at a particular region of Shank3, it was not clear whether CaMKII had other targets within Shank3. Based on our identification of S782 as a CaMKII site, we generated a phospho-state-specific antibody (pS782) to allow analyses of this phosphorylation *in situ* (Figs. 2, 3). We used this reagent and observed Shank3 S782 phosphorylation by CaMKII in a cellular context, which supports S782 site-specific phosphorylation by CaMKII. Although the phosphoantibody has a limited affinity to analyze endogenous Shank3 S782 phosphorylation in neurons and brain lysates, we demonstrated that CaMKII phosphorylates Shank3 on S782 *in situ* using cell lines.

Although we did not identify additional ASD-associated Shank3 variants that correspond with newly detected phosphorylation sites, we did examine the effect of an ASD-associated variant on S782 phosphorylation. Wang and colleagues characterized Shank3 S686 as a PKA phosphorylation site and investigated the effect of the *de novo* S686I ASD-associated variant on synapse and dendritic spine development (Wang et al., 2020). The *de novo* mutation in an ASD patient, Shank3 S686I, is several amino acids away from S782 and thus was a candidate to exert a potential effect. We evaluated this point mutation and observed an increase in S782 phosphorylation *in situ* without a robust change in the CaMKII association (Fig. 3*B*,*C*). However, we did not see any changes in Shank3 S782 phosphorylation in the samples from *in vitro* kinase assays with GST-Shank3 (473–903) WT and S686I fusion proteins (Fig. 3*C*). This discrepancy points to important factors within the *in situ* context that regulate Shank3 S782 phosphorylation. All these findings show that the ASD-associated Shank3 S686I mutation can affect the nearby CaMKII-mediated S782 phosphorylation, which indicates that dysregulation of the S686 and S782 phosphorylation may result in a combined pathologic mechanism for the ASD development.

To assess the effect of S782 phosphorylation, we first tested whether S782 mutations would affect the Shank3 binding with its well-known PDZ domain binding partners (Uchino et al., 2006; Jiang and Ehlers, 2013). In our results, S782 mutations did not alter Shank3 binding with GKAP or AMPARs in biochemical assays (Fig. 4). Notably, two previous studies found that disruption of Shank3 S685 phosphorylation specifically abolished Shank3-ABI1 binding, but did not alter GKAP, cortactin, L-type Ca^2+^ channel Ca_v_1.3, or Homer binding (Wang et al., 2020; Perfitt et al., 2020a). Along with these previous findings, our results (Fig. 4) suggest that S782 and S685 are within a region that is not a major regulatory site for Shank3 binding with Homer, cortactin, and other PDZ domain binding partners. More accurate tertiary protein structure of the Shank3 region is required for a better understanding of conformational regulation of relevant protein-protein interactions.

Because Shank3 is a critical scaffolding protein central to the organization of excitatory synapses, we assessed any potential effect of S782 on Shank3 enrichment and synapse size. Using immunofluorescence microscopy, we observed that expressing Shank3 that cannot be phosphorylated on S782 (S782A) increased both Shank3 and PSD-95 synaptic puncta size in dendritic spines compared with WT expression (Fig. 5*A–C*). It has been known that Shank3 and GKAP binding is important for Shank3 synaptic localization and that Shank3 overexpression has a positive correlation with PSD-95 clustering (Naisbitt et al., 1999; Han et al., 2013). However, because S782 phosphorylation did not affect binding to PDZ proteins, the increased Shank3 S782A and PSD-95 synaptic puncta size is unlikely to be related with GKAP binding (Fig. 4; Perfitt et al., 2020a; Wang et al., 2020). Pharmacological activation of global CaMKII in cultured neurons did not change colocalization between endogenous Shank3 and PSD-95 (Extended Data Fig. 5-1). Since CaMKII is known to phosphorylate multiple targets in synapses, it would be difficult to dissect S782 phosphorylation-specific effect of CaMKII pharmacological activation, but these results may imply that S782 phosphorylation specifically but partially regulates the association of Shank3 and PSD-95 in neurons.

As the ASD-associated Shank3 S685I mutation increases Shank3 S782 phosphorylation (Fig. 3*B*), we also examined the effect of S685I on Shank3 enrichment and synapse size. Interestingly, Shank3 S685I showed comparable synaptic puncta size to Shank3 WT, similar to Shank3 S782D (Fig. 5*A*,*C–E*). However, Shank3 S685I reduced PSD-95 puncta size without affecting colocalization with PSD-95 (Fig. 5*D*,*E*). From these results, we conclude that dephosphorylation of Shank3 S782 (S782A) increases Shank3 synaptic enrichment, whereas phosphorylation of Shank3 S782 (S782D) by CaMKII or following the S685I mutation retains Shank3 enrichment and synapse size. Importantly, Shank3 interacts with various synaptic actin signaling molecules, such as α-fodrin, ABI1, and Abp1 for actin nucleation and WASP family verprolin homologous protein (WAVE) complex (Böckers et al., 2001; Qualmann et al., 2004). These interactions are important for spine formation and maturation, and phosphorylation of the ASD-associated residue, S685, is involved in actin regulation (Kuriu et al., 2006; Wang et al., 2020). Dynamic and differential Shank3 remodeling in the PSD by neuronal activity is highly dependent on the actin cytoskeleton (Kuriu et al., 2006; Tao-Cheng et al., 2016). Since neuronal activity-dependent translocation of CaMKII to the PSD is also well characterized (Shen and Meyer, 1999; Tao-Cheng, 2020), Shank3 S782 may be a CaMKII regulated phosphorylation site that balances the Shank3 association with the actin signaling molecules that are regulated by Shank3 S685 phosphorylation. Additional experiments are required to understand more about the possible mechanistic link between these proteins.

In this study, we characterized CaMKII phosphorylation of Shank3 S782 using a variety of assays. Although S782 does not show up in databases as an ASD-associated variant, we did find that Shank3 S782 phosphorylation is affected by the nearby ASD-associated variant, S685I. Furthermore, we found that this variant is involved in Shank3 synaptic enrichment. Because both CaMKII and Shank3 play such critical roles at excitatory synapses during development and activity-dependent plasticity, we think this newly identified regulatory site is worthy of future studies involving *in vivo* mouse models.

## Synthesis

Reviewing Editor: Isabel Perez-Otaño, Instituto de Neurociencias Decisions are customarily a result of the Reviewing Editor and the peer reviewers coming together and discussing their recommendations until a consensus is reached. When revisions are invited, a fact-based synthesis statement explaining their decision and outlining what is needed to prepare a revision will be listed below. The following reviewer(s) agreed to reveal their identity: Carlo Sala, Johannes Hell. Note: If this manuscript was transferred from JNeurosci and a decision was made to accept the manuscript without peer review, a brief statement to this effect will instead be what is listed below.

I am pleased to inform you that your paper, “CaMKII phosphorylation regulates synaptic enrichment of Shank3,” has been judged potentially suitable for publication in eNeuro, provided appropriate revisions are made.

Your paper was reviewed by two reviewers who found your manuscript to be of significance and the data of high quality, but indicated the need for revisions. Particularly, they would like to see controls validating the specificity of the antibody and functional experiments. Before we can make a final decision about publication, we will have your paper re-reviewed by one or both of the reviewers. The reviews are appended to this email.

<b>

In this paper the authors studied the basal and activity-dependent phosphorylation of Shank3 complementary approaches such as mass spectrometry analysis, biochemical assays, and expression in cultured neurons.

They mainly identified serine 782 (S782) as a potent CaMKII phosphorylation site, demonstrated also using a phosphorylation state-specific antibody.

Interestingly they also observed that the ASD-associated variant (Shank3 S685I), induces increased S782 phosphorylation.

Finally, they showed that eliminating phosphorylation of Shank3 with a S782A mutation increased Shank3 and PSD-95 synaptic puncta size without affecting Shank3 colocalization with PSD-95 in cultured hippocampal neurons.

- The paper presents convincing data demonstrating the phosphorylation of Shank3 in the S782 by CaMKII, however, the role of this phosphorylation in regulating Shank3 accumulation to synapses is not very strong. Indeed the accumulation of Shank3 S782A in comparison to WT or S782D might depend also on the expression level of the different constructs. Thus the data will be more strong if the authors will try to look at the endogenous Shank3 accumulation upon CaMKII pharmacological activation or inhibition or after transfection in neurons of constitutively active CaMKII (CaMKII T286D) or inactive CaMKII (CaMKII R42K).

- Shank3 S685I variant, induces increased S782 phosphorylation, is this variant is less localized to synapses? This experiment will also help to better understand the role of S782 in Shank3 localization to synapses.

Reviewer 2

The authors identify through mass spectrometry S782 as a new phosphorylation site for CaMKII. Functional studies with phospho-deficient S782A and phospho-mimetic S782D mutant SHANK3 in hippocampal cultures suggest that S782 phosphorylation impairs postsynaptic PSD-95 accumulation.

Major concerns

1. It is unclear whether their phospho-specific antibody against pS782 is specifically recognizing this site or potentially other phosphorylation sites as well. It would be necessary to show that, for instance, mutating S782 to Ala leads to loss of immunoblot staining with this AB under conditions under which CaMKII phosphorylates this site.

2. Fig 4 and 5: that the phospho-mimetic S782D mutation does not alter SHANK binding to GKAP or AMPARs does not exclude that phosphorylation of S782 could affect this binding because Asp is anything but a perfect mimetic of a phospho-group. Some sort of functional effect is suggested in Fig 5 by the S782A mutation which augments postsynaptic PSD-95 even though the S782D mutation had not effect. A simple explanation is that the A mutation prevented basal phosphorylation but the D mutation does not mimic phosphorylation well enough to cause a detectable effect. The authors should test whether phosphorylation of S782 by CaMKII either before pull down or by co-expression in HEK cells affects GKAP interaction. It would be desirable to test a few other interactions here but I can see that this is too open ended and thus it may not be possible to be within the scope of the current work.
