# Supplementary figures and images for: CaMKII Phosphorylation Regulates Synaptic Enrichment of Shank3
Source: eNeuro. 2021 May 22;8(3):ENEURO.0481-20.2021. doi: 10.1523/ENEURO.0481-20.2021 (PMC8152369; doi:10.1523/ENEURO.0481-20.2021)

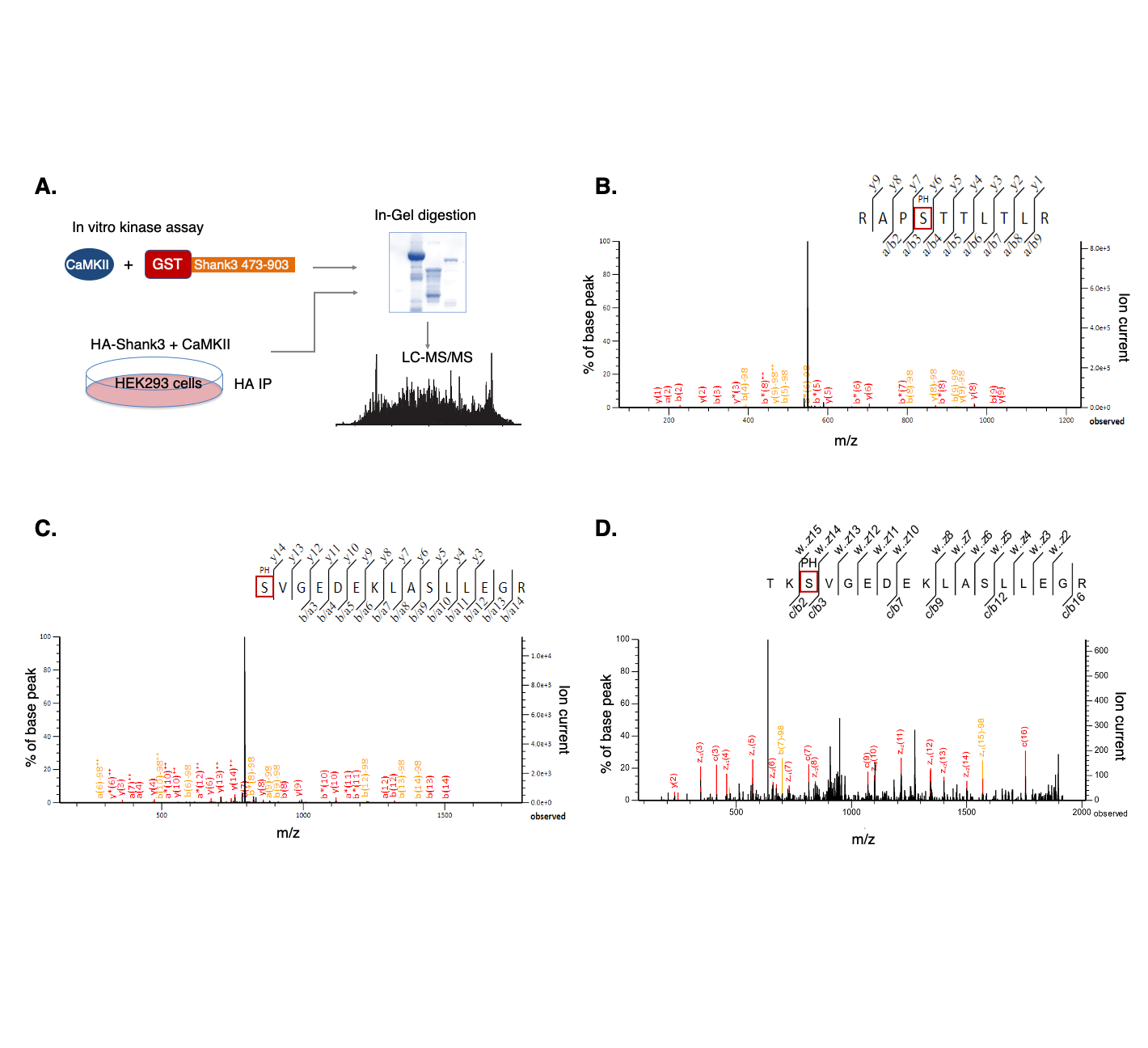

Supplement: Extended Data Figure 2-1 — MS analysis from in vitro phosphorylation assays and in situ experiments. A, A schematic of the experimental procedure for the analysis of the samples from in vitro phosphorylation assays and in situ experiments. B, C, Representative MS/MS spectra of the Shank3 S685 and S782 phosphorylated peptide found in the samples from in vitro phosphorylation assays. D, Representative MS/MS spectrum of the Shank3 S782 phosphorylated peptide found in the samples from in situ experiments. Download Figure 2-1, TIF file. [file enu-eN-NWR-0481-20-s03.tif]

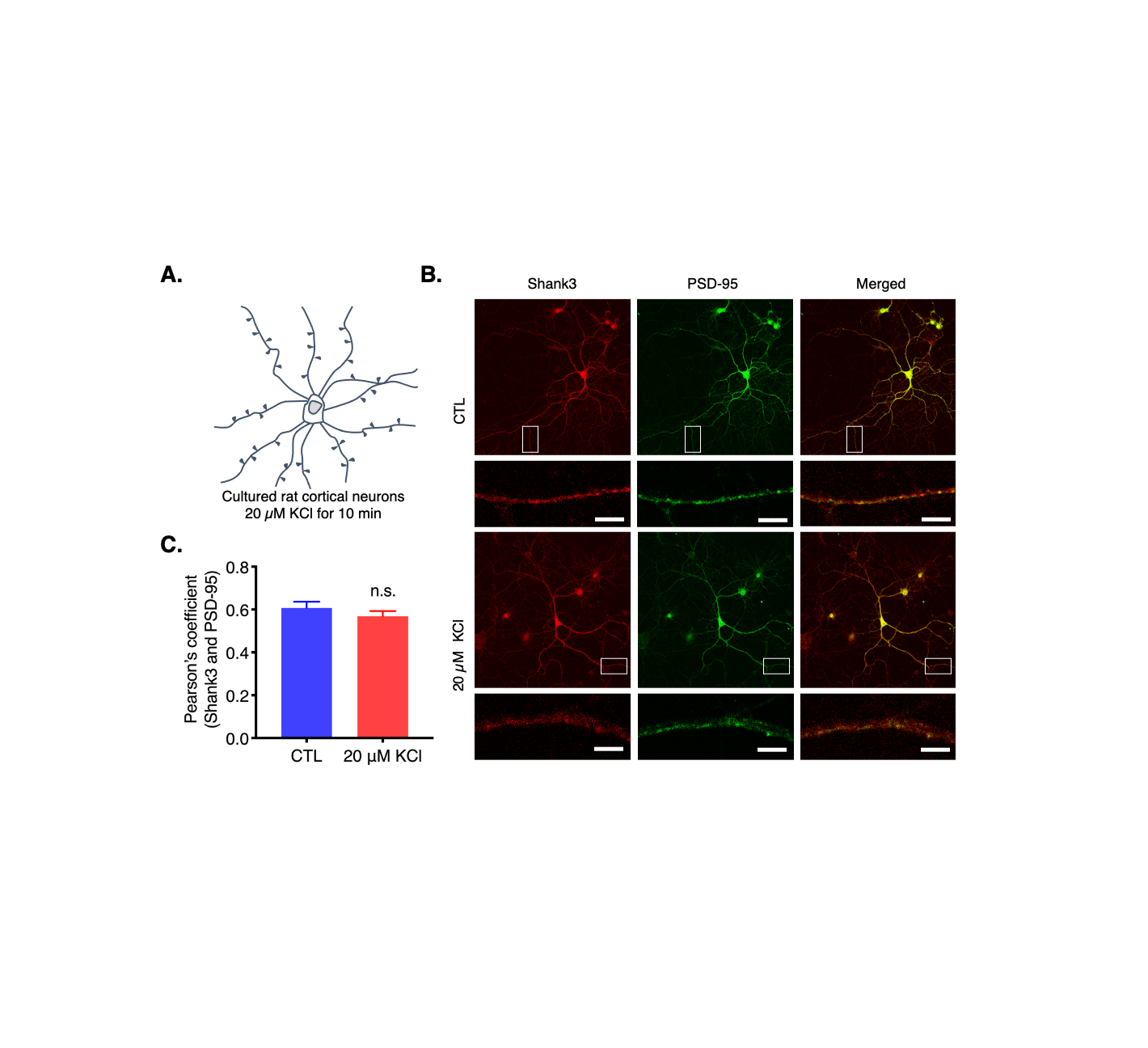

Supplement: Extended Data Figure 5-1 — No detectable effect of CaMKII pharmacological activation on the colocalization between endogenous Shank3 and PSD-95 in neurons. A, B, Cultured rat cortical neurons were treated with 20 μm KCl for 10 min to activate endogenous CaMKII. Endogenous Shank3 was stained with anti-Shank3 and Alexa Fluor 555-conjugated secondary antibody (red). Endogenous PSD-95 was labeled with anti-PSD-95 antibody and Alexa Fluor 488-conjugated secondary antibody (green). Regions from the secondary dendrites are shown. Scale bar: 10 μm. C, Regions from the secondary dendrites were analyzed for Pearson’s coefficient. Graph indicates mean ± SEM (n = 10 for CTL, n = 10 for KCl treatment). Statistics using an unpaired t test. n.s., not significant. Download Figure 5-1, TIF file. [file enu-eN-NWR-0481-20-s01.tif]
